# Of tests, trochs, shells, and spicules: Development of the basal mollusk *Wirenia argentea *(Solenogastres) and its bearing on the evolution of trochozoan larval key features

**DOI:** 10.1186/1742-9994-7-6

**Published:** 2010-01-26

**Authors:** Christiane Todt, Andreas Wanninger

**Affiliations:** 1 Department of Biology, University of Bergen, Thormøhlensgate 53a, N-5008 Bergen, Norway; 2 Research group for Comparative Zoology, Department of Biology, University of Copenhagen, Universitetsparken 15, DK-2100 Copenhagen, Denmark

## Abstract

**Background:**

The phylogenetic status of the aplacophoran mollusk taxon Solenogastres (Neomeniomorpha) is controversially discussed. Some authors propose the clade to represent the most basal branch within Mollusca, while others claim aplacophoran mollusks (Solenogastres and Caudofoveata) to be derived. Larval characters are central in these discussions, specifically the larval test (calymma, apical cap), the ontogeny of the epidermal scleritome, and the proposed absence of larval protonephridia. To date, developmental data are available for five solenogaster species, but most reports are incomplete and need confirmation.

**Results:**

*Wirenia argentea *deposit small batches of uncleaved embryos that are tightly enclosed by a smooth and transparent egg hull. Cleavage is spiral and unequal. The ciliated larvae hatch about 45 hours after deposition and swim actively in the water column. Within 48-60 hours after hatching they become mushroom-shaped with a pronounced apical cap partly enclosing a posterior trunk. The cells covering the apical cap are large and cleavage arrested. On the apical cap there is a prominent prototrochal band of compound cilia and an apical ciliary tuft and the trunk bears a terminal ciliary band (telotroch). Obscured by the apical cap, a ciliary band originates in the stomodaeal pore and surrounds the trunk. As development is proceeding, the trunk elongates and becomes covered by cuticle with the exception of a ventral ciliary band, the future foot. The larvae have a pair of protonephridia. At 5 days after hatching they begin to settle and within the following 7-9 days the apical cap is gradually reduced. Scattered epidermal sclerites form under the cuticle. *Wirenia argentea *lack iterated groups of sclerites at any developmental stage. At 40 days after hatching, the postlarvae have a fully developed foregut, but the midgut and hindgut are not yet interconnected.

**Conclusions:**

Solenogastres develop via a trochophore-like lecitotrophic larva with a preoral apical cap that at least partly represents an enlarged prototrochal area. Homology of this larval type (pericalymma larva) to test cell larvae of other spiralian clades is doubtful. The ontogeny of *W. argentea *does not provide any evidence for a derived status of Solenogastres within Mollusca.

## Background

**Figure 1:**
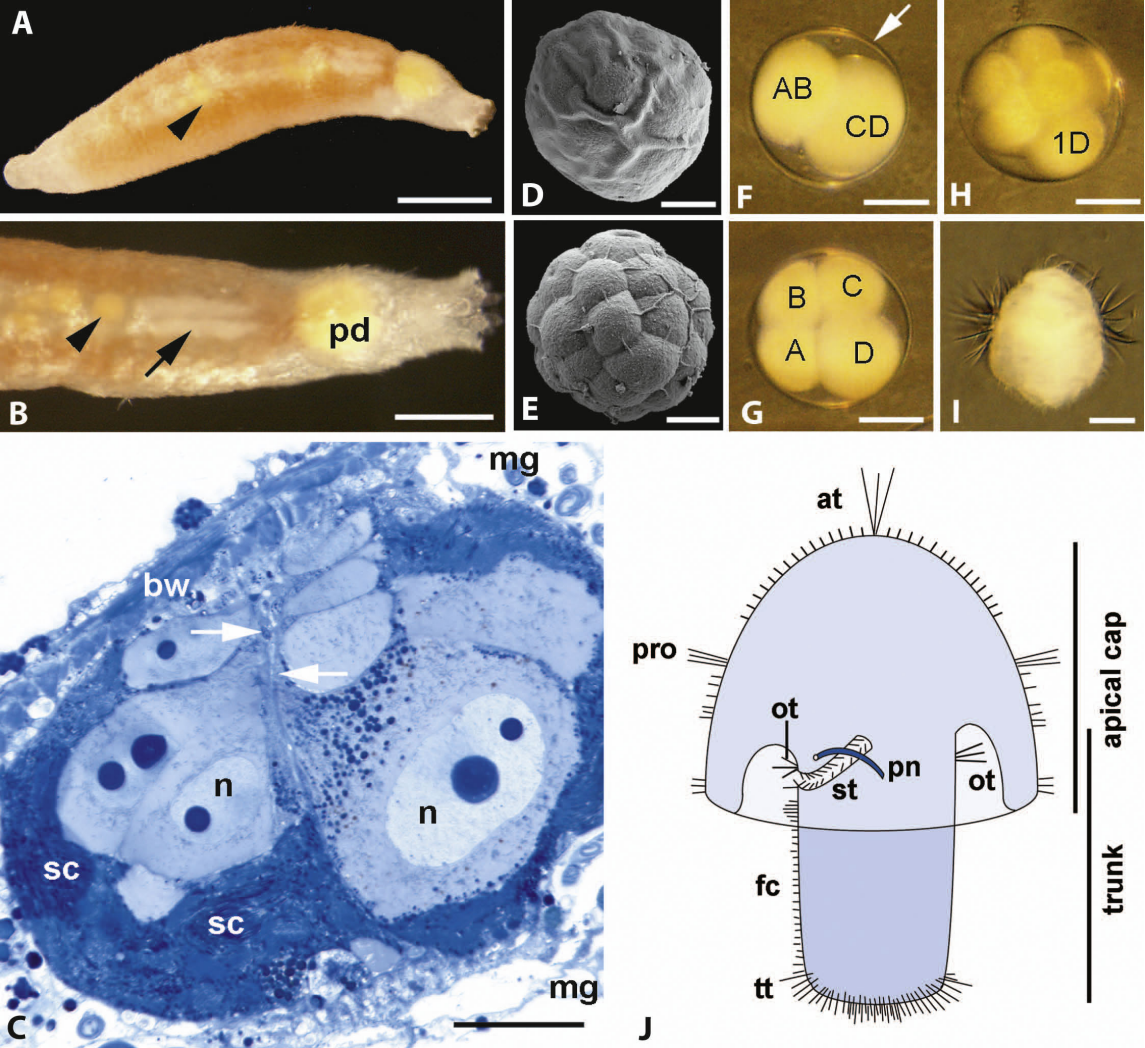
**Oocytes, early development, and gonad structure in *Wirenia argentea***. A, B. Adult specimen bearing eggs in the gonad (arrowheads) and pericardium (pd). A. Scale bar 500 μm; B. Arrow indicating posteriormost part of the gonad containing white sperm, scale bar 250 μm; C. Gonad of *W. argentea*, histological cross section. Oocytes of different developmental stages with large nuclei (n) containing two dark-staining nucleoli are attached to a median diaphragm (arrows) and surrounded by spermatogonia (sc); bw: body wall, mg: midgut; scale bar 50 μm; D, E. Scanning electron micrographs of an uncleaved embryo (D) and a 32 cell stage (E) covered by the smooth egg hull, scale bars 30 μm; F-H. Light micrographs of 2-cell stage (F), 4-cell stage (G), and 8-cell stage (H), macromeres conventionally marked, the arrow in F points to the egg hull. Note that the CD, D and 1D blastomeres are larger than the other blastomeres; scale bar 40 μm; I. Larva 12 hours after hatching, light micrograph, scale bar 40 μm; J. Schematic drawing of a pericalymma larva depicting apical cap and trunk, stomodaeum (st), protonephridium (pn), as well as the main ciliary bands; at: apical tuft, fc: foot ciliation, ot: oral troch, pro: prototroch, tt: telotroch.

The Solenogastres (Neomeniomorpha) supposedly constitute the most basal extant mollusk lineage [1-3]. Suggested plesiomorphic characters include a mantle covered with cuticle and calcareous sclerites (spicules), a simple radula without subradular membrane, the lack of a midgut gland, the general structure of the nervous system, and the presence of oblique musculature in the body wall [4-6]. Solenogastres are known to develop via a so-called test cell larva [(= pericalymma larva *sensu *Salvini-Plawen [7]; pericalymma Type 1 *sensu *Nielsen [8]], a type of lecithotrophic larva first described for protobranch bivalves [9,10]. Characteristic for test cell larvae is a cap-like cover (larval test) of large ciliated cells enveloping at least part of the larval body (Fig. 1). Cilia may be scattered, but more often they are arranged in distinct ciliary bands. Based on the presence of a comparable larval type in certain polychaetes and sipunculans, the pericalymma larva has been interpreted as the ancestral larval type of mollusks and even of all spiralians [7,11], a view strongly refuted by others [[12], for review].

The ancestral status of Solenogastres within Mollusca is not undisputed [13-15], with some authors proposing that the body organization of aplacophoran mollusks, i.e. Solenogastres and their putative sister group, Caudofoveata (Chaetodermomorpha), is the result of simplification and character loss. Based on molecular data, this notion has recently been brought up again [16]. Some authors propose a shell-bearing chiton- (i.e., polyplacophoran)-like ancestor as basal for Mollusca, a hypothesis claimed to be supported by ontogenetic data showing a serial arrangement of dorsal epidermal sclerites in species of Solenogastres [17,18] and Caudofoveata [19]. The larval/postlarval sclerite rows have been suggested to represent rudiments of spicule rows separating reduced serial dorsal shell fields [18]. Others [20,21] interpreted the serially arranged sclerites as possible precursors of the seven shell plates found in recent postmetamorphic polyplacophorans. Seven dorsal rows of sclerites seem in fact to occur in larvae of the caudofoveate *Chaetoderma *sp. [19], but the observation of seven rows of large imbricating scales covering the dorsal surface of a solenogaster larva (*Nematomenia banyulensis*) was based on a single specimen [17] and thus requires reinvestigation. Two postmetamorphic specimens of an unidentified solenogaster species have been described, where the younger specimen showed six distinct sclerite-covered dorsal areas [18]. In larvae of *Epimenia babai*, by contrast, epidermal sclerites emerge scattered over the entire dorsal surface without showing any seriality [22,23]. To evaluate these contradictory findings, data on additional solenogaster species are crucial.

We studied the ontogeny of *Wirenia argentea*, a solenogaster species classified within the Gymnomeniidae (Pholidoskepia), a clade supposedly representing an early branch within Solenogastres [5]. These animals are abundant on silt and mud in water depths of about 150-600 m in the northern European Atlantic and for the present study specimens were kept in the laboratory for several months. We describe behavioral aspects of oviposition and larval locomotion and focus on embryonic, larval and postlarval development, taking account of external morphology as well as of selected aspects of organogenesis. Our results shed new light on the ancestral organization of mollusk larvae and allow for a reevaluation of evolutionary relationships among spiralian test-cell larvae.

## Results

### Oogenesis, fertilization, and egg deposition

In adult living animals with the body wall musculature not overly contracted, the individual oocytes in the gonad are visible through the dorsal body wall as paired, light yellowish globules. The minimum size limit for reproduction is about one third of the maximum body length, but large animals (8-9 mm body length) produce considerably more eggs. Four to six ripe eggs are stored tightly packed in the pericardium for several hours before deposition (Fig. 1A, B). The posteriormost part of the gonad in specimens bearing ripe eggs generally appears as paired whitish (containing sperm?) structure (Fig. 1B). From histological sections of fully mature specimens it becomes obvious that sperm and eggs are produced simultaneously (Fig. 1C).

Nearly all eggs deposited in the laboratory were fertilized and underwent first stages of cleavage, even if some showed irregularities later in development. Copulation was not observed and had probably taken place in the natural habitat prior to sampling. Self-fertilization is also a possibility, especially because the seminal vesicles of several specimens depositing fertilized eggs in the laboratory contained hardly any sperm.

During egg deposition the animals lift the posterior part of the body (ca. 1/3 of total body length) and expand their posteriorly located pallial cavity opening to release two eggs, one after the other; after some minutes the next two eggs are released, then in large specimens two more. Six eggs appear to be the maximum in one session. It takes 24 hours or more for the next batch of eggs to be ready for deposition. Transport of the rather large eggs through the spawning ducts appears to be due to muscular activity, as rhythmic peristaltic waves occur during the process.

The fertilized eggs are globular, measure 100 μm in diameter, and bear a clear, smooth, slightly sticky egg-hull covered by a thin layer of mucus (Fig. 1D-I). The eggs are oval in shape immediately after release, but they become globular within two to three minutes. First they float, but in a dish with stagnant water they sink to the bottom within about fifteen minutes and then stick to the substrate (detritus as well as glass or plastic).

### Cleavage

At 7°C, the first cleavage is completed within three hours after deposition of the fertilized eggs (Fig. 1F). Subsequently, the cells divide every two to three hours, until a 32-cell stage is reached (Fig. 1E). The first cleavage is slightly unequal, with the larger CD blastomere bearing a large polar lobe, which appears as a whitish to semitransparent bulge on the surface (Fig. 1F). After the second cleavage, the A, B, and C quadrants are of equal size, while the D quadrant is larger (Fig. 1G). After the second cleavage a second polar lobe, similar in appearance to the first one but smaller, appears on the D quadrant. The 1D cell of the 8-cell stage is conspicuously larger than all other cells (Fig. 1H). Cleavage is spiral and unequal, but in later stages there are no clear size differences between macromeres and micromeres (Fig. 1E). Gastrulation takes place 25-27 hours after egg deposition. About four hours later the embryos start to rotate within the egg-hull, a movement caused by ciliary action, which can be observed through the still entirely transparent egg-hull. At this stage, the embryos are roughly globular but flattened at the posterior pole. Within the next 5-10 hours they become slightly elongated and the pseudo-blastopore - the opening of the developing apical cap - comes to lie in a ventro-posterior position.

### Hatching and larval behavior

**Figure 2:**
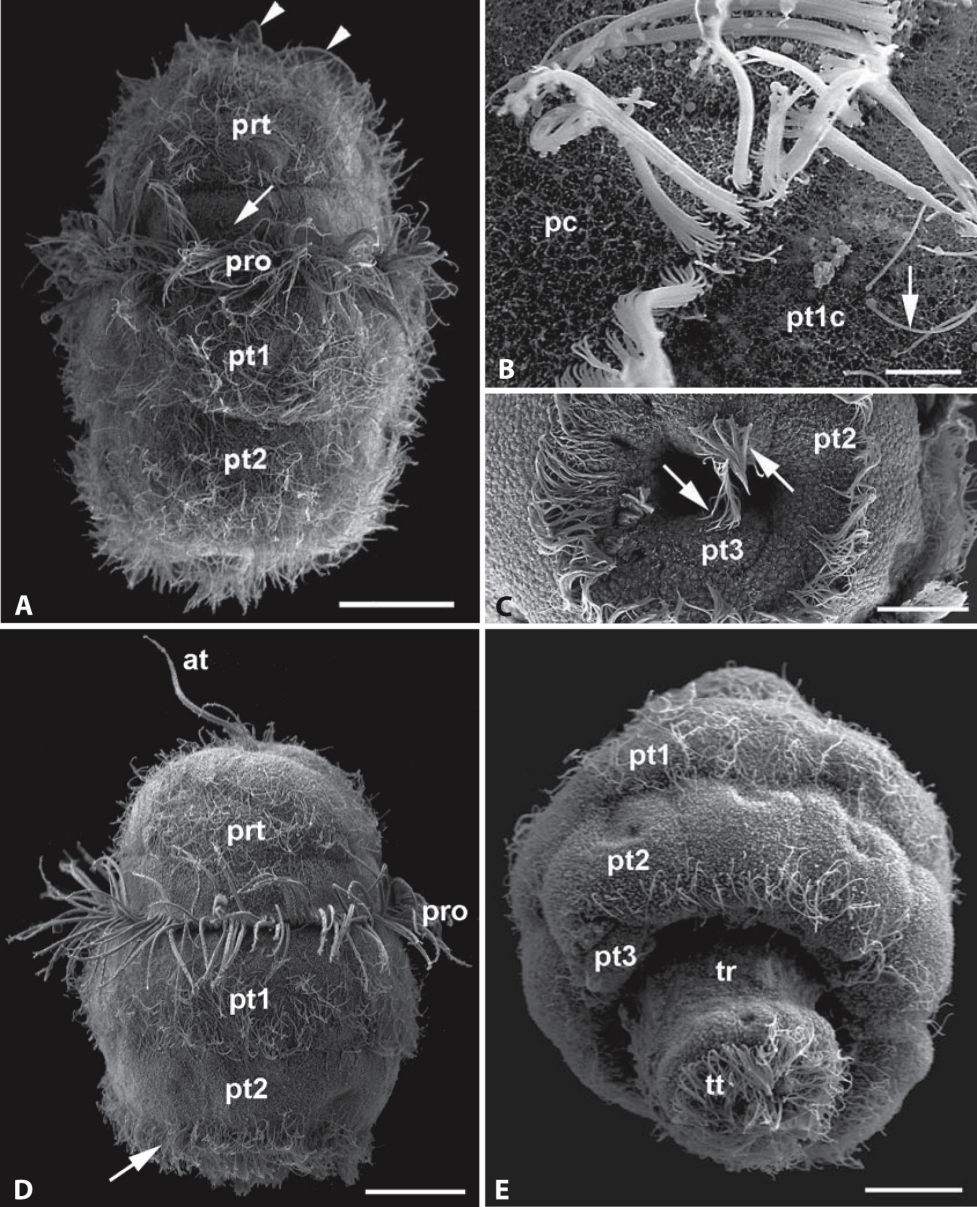
***Wirenia argentea *larvae, day 1-3 after hatching, SEM micrographs**. A. Newly hatched larva, apical cap covered by a single row of prototrochal cells bearing compound prototroch cilia (pro), two rows of posttrochal cells (pt1, pt2), and pretrochal cells (prt). The compound cilia of the apical tuft (at) are bent. Note the cilia-free surface above the prototrochal ciliary band and the dense ciliation of the posttrochal cell rows (arrow), scale bar 30 μm; B. Detail of prototroch with compound prototroch cilia on the posterior margin of the prototroch cells (pc). Note scattered cilia (arrow) on the adjacent posttrochal cells (pt1c), scale bar 4 μm; C. Detail of pseudo-blastopore, seen from below, showing the second row of posttrochal cells (pt2) and the two posteriormost test cells (pt3) bearing tufts of cilia (arrows), scale bar 15 μm; D. Day 2 larva with long apical tuft (at) and conspicuous prototroch cilia (pro). Pretrochal cells (prt) and first row of posttrochal cells densely ciliated, second row of posttrochal cells with a posterior ciliary band (arrow); scale bar 30 μm; E. Day 3 larva in oblique adapical view with trunk (tr) and telotroch (tt) protruding from larval test; pt1-pt3: posttrochal cell rows 1-3, scale bar 20 μm.

The larvae hatch at about 45 hours after oviposition. During this process, they move actively, colliding with the egg hull and thereby extending and thinning the hull gradually until it breaks. Several small refractile globules that may contain enzymes to soften the egg-hull are floating between larva and egg-hull. Once the egg-hulls are broken, the larvae (Figs. 1I, 2A) escape rapidly and start to swim upwards. During swimming they rotate slowly clockwise around their longitudinal axis. The swimming movements appear to be mostly due to action of the long and thick compound cilia of the main prototrochal ciliary band, while the apical ciliary tuft, composed of 3-5 compound cilia, moves only slowly. The larvae do not react to a light source positioned laterally or above the culture dish. They are negatively geotactic: In a glass cylinder with stagnant water a maximum vertical swimming distance of 30 cm from the bottom could be observed. When disturbed, many of the young larvae stop swimming and sink to the bottom but start swimming up again after some minutes. Within 48-60 hours after hatching, the still swimming larvae elongate and develop the typical mushroom-shaped body with an apical cap partly covering a posterior trunk (Fig. 1I). Settling is not induced by a specific substratum. When kept at 7°C, larvae begin to settle spontaneously at about 5 days after hatching and then complete metamorphosis within a week. The transition from planktonic to benthic lifestyle is gradual (for morphological details see below), with larvae spending increasingly more time close to or on the bottom of the dish but keeping their ability to swim for another four to five days. Upon settling, the apical caps comprise about one third of the larvae's total body length. The larvae move slowly over the bottom of the dish and their tests become reduced in size. From the point of settling onwards, the foot cilia gradually take over the locomotory function. Epidermal sclerites appear about two weeks after hatching, at a stage where the apical cap still is partly present and the prototroch functional. At this stage, the larvae are not able to swim up into the water column, but the prototroch cilia may cause a rotating movement, with the posterior tip of the trunk on or very close to the bottom of the dish. At about 2 weeks after hatching, the apical cap is completely reduced. Superficially, the young postlarvae resemble miniature adults, but they are yellowish in color (instead of pink) and the shape of epidermal sclerites diverges from that of adult specimens (see below). At 42 days after hatching, the yellowish yolk is almost completely used up and the young juveniles are white and slightly translucent. All remaining juveniles were fixed at this stage.

### Larval morphogenesis

#### Day 1-2 after hatching

**Figure 3:**
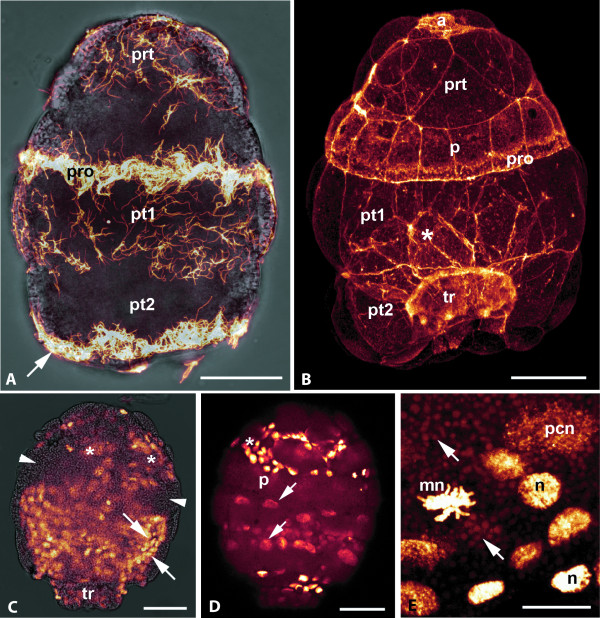
***Wirenia argentea *larvae, day 1-3 after hatching, anti-α tubulin labeling, overlay of confocal image stacks**. A, B. Day 1 larva. A. Overlay of CLSM maximum projection (anti-α-tubulin labeling) and light micrograph. Note strong signal from prototrochal (pro) ciliary band and cilia (arrow) of second posttrochal cell row (pt2). Pretrochal cells (prt) and cells of first posttrochal row (pt1) bear scattered cilia; B. Phalloidin labeling showing areas of high actin content (cell borders, cilia); note irregular size of pretrochal (prt) and central cells (asterisk) and regular arrangement of prototrochal (p) and posttrochal (pt1, pt2) cells; a: apical organ, pro: prototrochal ciliary band, tr: trunk rudiment; scale bar 35 μm; C, D. Day 3 larva in lateroventral view, scale bars 50 μm; C. Overlay of Hoechst nuclear staining and light micrograph, outermost cell layer of apical cap omitted, thus showing small cell nuclei of mesodermal cells and ectodermal cells of the trunk (tr) and apical cap inner surface (arrows); note ring-shaped zone devoid of nuclei (arrowheads) and cerebral ganglion anlagen (asterisks), D. Hoechst nuclear staining, showing nuclei of outermost cell layers; note size difference of posttrochal cell tier nuclei (arrows) and cerebral depression nuclei (asterisk); nuclei of prototroch cells (p) only faintly labeled. E. Detail of outermost cell layers of apical cap of a Hoechst-labeled three day old larva showing a large prototroch cell nucleus (pcn), several smaller mesoderm nuclei (n), including one in mitosis (mn); note dense yolk granules (arrows); scale bar 12 μm.

Upon hatching, the larvae are ovoid in shape with a pronounced prototrochal ciliary band (prototroch *s. str*.) and a long apical ciliary tuft (Fig. 1I, 2A). After 24 hours the larval body becomes more bell-shaped with a blunt caudal end (Fig. 2D, 3A, B). The prototroch cells bear a single row of compound cilia (cirri) that are composed of about 30 locomotory cilia each (Fig. 2B). The remaining surface is devoid of cilia but covered by microvilli (Fig. 2A, B). The pretrochal cells have a roughly triangular outline and are arranged in 2-3 rows (Fig. 3B). They bear scattered cilia and a dense coat of microvilli. The apical tuft is composed of 3-5 cirri, but the number of cells underlying the apical tuft is unknown. Posterior to the prototroch, there are two regular tiers of large cells bearing scattered cilia that in older larvae tend to be more densely spaced towards the posterior cell margin, especially in the second (posterior) tier. The cells of the anterior post-trochal tier are about twice the size of the prototrochal cells and somewhat larger than the cells of the posterior tier. There is a pair of cells with tufts of cilia bordering the posterior apical cap opening (Fig. 2C). Specific characteristics of the ciliation of test cell types are given in Table 1. All cilia have a long vertical and a shorter horizontal rootlet.

**Table 1:** Apical cap of *Wirenia argentea*, test cell numbers and characteristics

**Cell position** **(tier)**	**Number of** **cells/row**	**Number of** **cilia/cell**	**Length of** **cilia (μm)**	**Arrangement** **of cilia**		
		
						
		
Apical	?	3 × (15-20)*	40-50	tuft		
		
Pretrochal 1	6-8	numerous	10-15	scattered		
		
Pretrochal 2	8-10	numerous	10-15	scattered		
		
Prototrochal	14-16	6 × (20-25)*	25-35	adapical row		
		
Posttrochal 1	10-12	numerous	10-15	scattered		
		
Posttrochal 2	12-14	numerous	10-15	adapical row		
		
Posttrochal 3	2	30-40	10-15	tuft		

* Arranged as compound cilia (cirri).

The test cells covering the apical cap are considerably larger than all other larval cells and their nuclei are about four times the size of other cell nuclei (Fig. 3D, E). Test cell numbers remain constant until reduction of the cap after settlement, while other larval tissues show mitosis (Fig. 3E). The density of non-test nuclei is highest in the posteriormost part of the larva, where the trunk is formed, and in an anterior zone that probably is associated with formation of the central nervous system (Fig. 3C, D).

#### Day 3-7 after hatching

**Figure 4:**
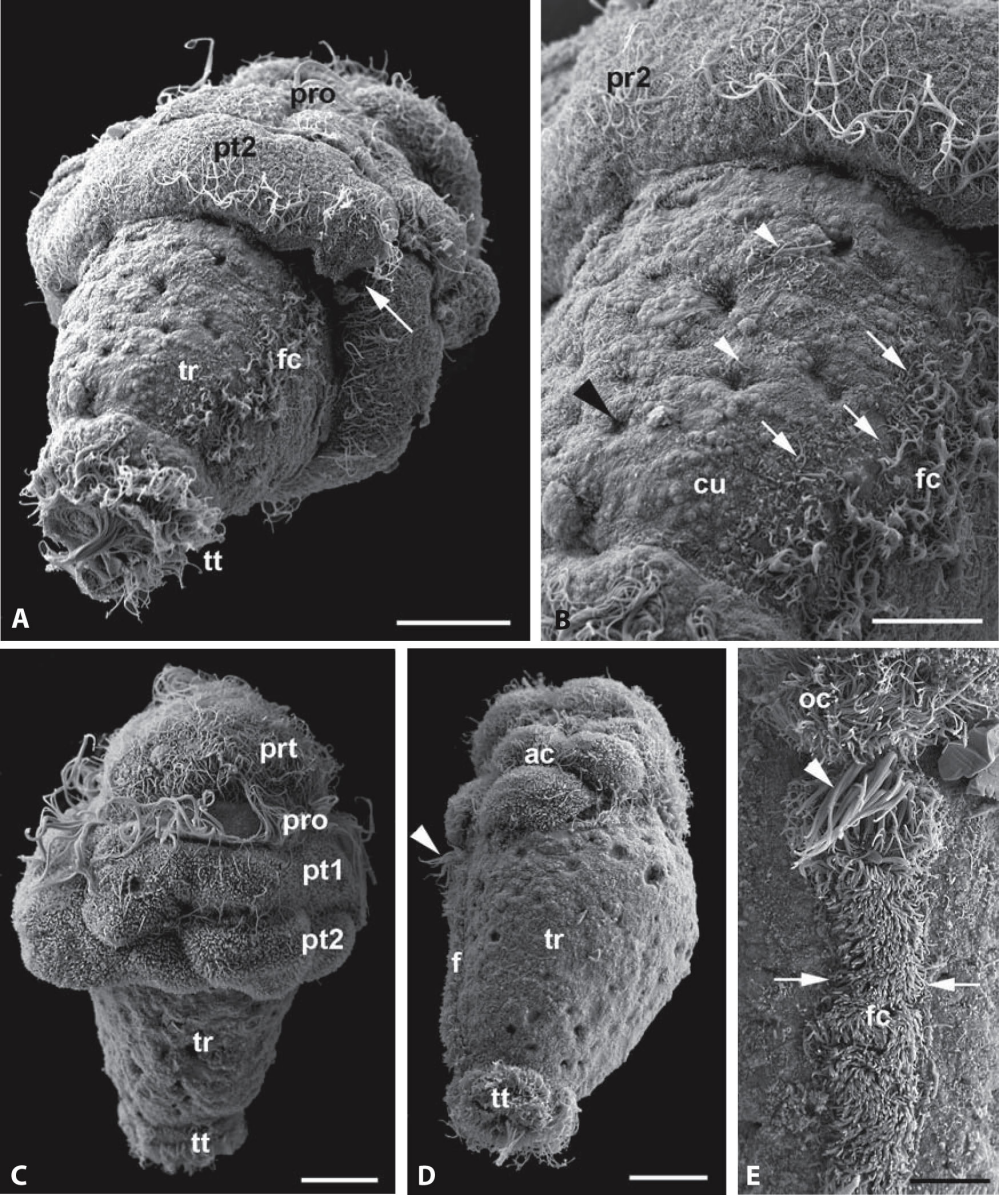
***Wirenia argentea *larvae (A-C) and postlarvae (D-E), SEM micrographs**. A. Day 5 larva, ventrolateral view. The trunk (tr) is largely exposed and bears foot cilia (fc) and a prominent telotroch (tt), the second tier of posttrochal cells (prt2) shows a ventral cleft (arrow); the prototroch cilia (pro) are inconspicuous due to a preparation artifact; scale bar 20 μm. B. Detail of figure A; the surface of the trunk is covered by cuticle (cu) and single scattered cilia (white arrowheads); note that there is no clear mantle margin (arrows) but that foot cilia (fc) are arranged irregularly; the small depressions (black arrowhead) may represent gland openings; scale bar 10 μm. C. Day 7 larva with exposed trunk (tr) bearing telotroch (tt) and large apical cap; note that the posttrochal cells (pt1, pt2) are irregular in shape and lack cilia, while the prototroch (pro) and pretrochal area (prt) are intact; scale bar 20 μm. D. Day 10 postlarva, early crawling stage in a lateral view; the foot (f) and prepedal ciliary pit (arrowhead) are fully developed, part of the apical cap (ac) and the telotroch are still present; scale bar 20 μm. E. Detail of anterior part of the foot with prepedal ciliary pit (arrowhead) and oral ciliation (oc); note the sharp border between mantle cuticle and foot ciliation (arrows); scale bar 10 μm.

**Figure 5:**
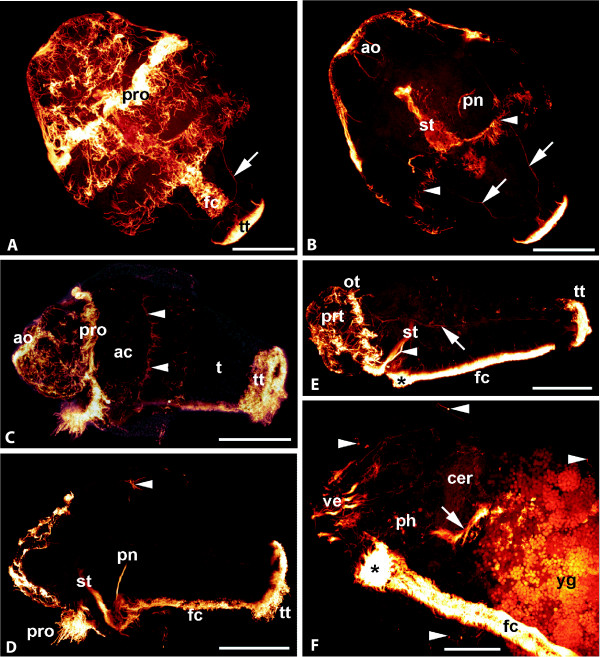
***Wirenia argentea *larvae and postlarvae (40 days), anti-α tubulin labeling, overlay of confocal images**. A, B. Day 4 larva, ventral view; scale bars 40 μm. A. Image stack through entire Z-axis showing prototrochal ciliary band (pro), telotroch (tt), foot cilia (fc), and longitudinal neurite bundles (arrow). B. Reduced image stack omitting outermost cell layers; note apical organ (ao) with associated nerves, paired longitudinal neurite bundle (arrows), stomodaeum (st), and protonephridia (pn); oral troch (arrowheads). C, D. Day 6 larva, lateral view; scale bars 50 μm. C. Overlay of light micrograph and confocal image; apical cap (ac) covers part of the trunk (t); apical organ with ciliary tuft (ao); prototrochal cilia (pro) and telotroch (tt) brightly labeled, oral troch faintly visible (arrowheads). D. Reduced image stack omitting outermost cell layers and layers below sagittal plane; dorsal part of oral troch (arrowhead); fc: foot cilia, pn: protonephridium, pro: prototroch cilia, st: stomodaeum, tt: telotroch. E. Day 11 postlarva, crawling stage; pretrochal part of apical cap (prt) still present, oral troch (ot) exposed; foot cilia (fc) and prepedal ciliary pit (asterisk); note lateral longitudinal neurite bundle (arrow); st: stomodaeum, tt: telotroch; scale bar 50 μm. F. Anterior end of a postlarva 30 days after hatching; cilia present in the gut (arrow), but distal pharynx (ph) devoid of cilia; prepedal ciliary pit marked by asterisk, sensory nerve endings by arrowheads; cer: cerebral ganglion, fc: foot cilia, ve: vestibular cirri, yg: yolk granules; scale bar 25 μm.

**Figure 6:**
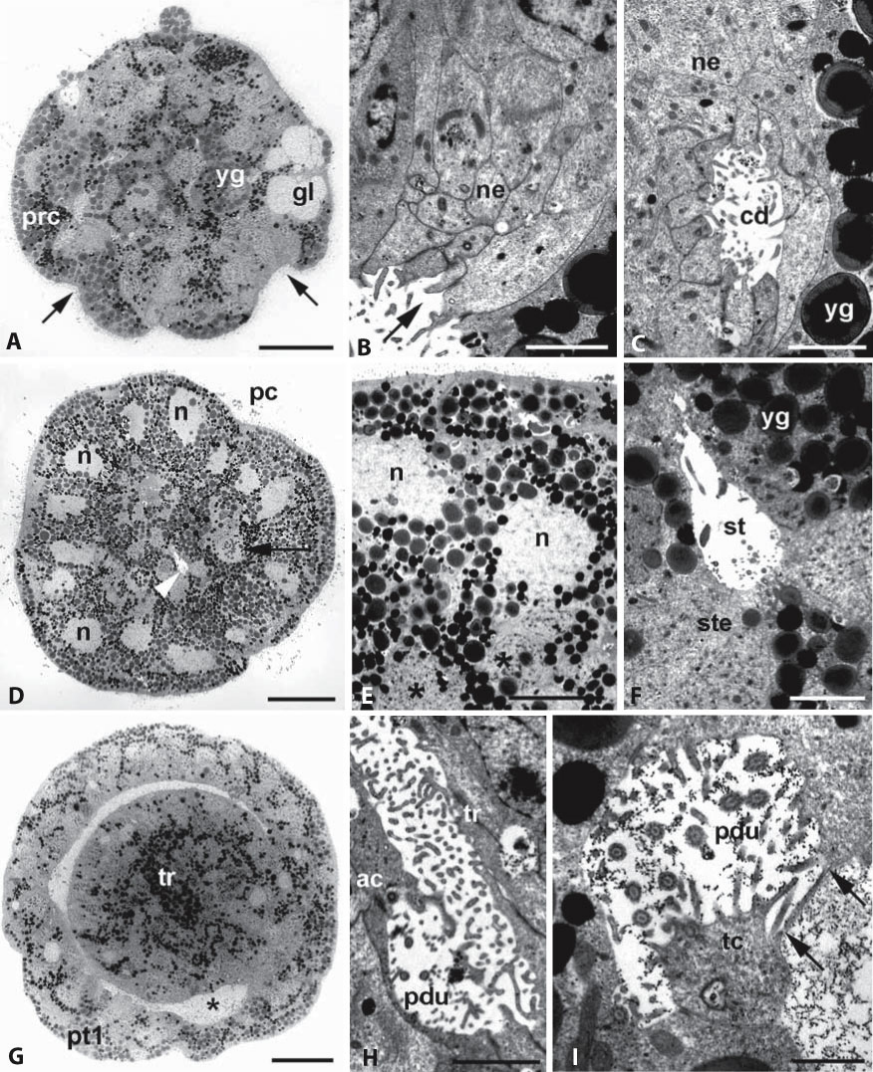
***Wirenia argentea *larva at day 7, TEM micrographs of cross sections through the apical cap**. A. Section anterior to prototroch depicting cerebral depressions (arrows), pretrochal cells (prc), and gland cells (gl); yg: yolk granules; scale bar 20 μm. B. Detail of A; surface of cerebral depression with microvilli (arrow); ne: neurons; scale bar 2 μm. C. Lumen of cerebral depression (cd) surrounded by neurons (ne); yg: yolk granule of pretrochal cell; scale bar 2 μm. D. Section through prototrochal plane; note compound prototrochal cilia (pc), large nuclei of prototroch cells (n), stomodaeum (arrowhead), and mesodermal cell with nucleus in mitosis (arrow); scale bar 20 μm. E. Detail of D, nuclei of prototrochal cells (n) larger and more electron-translucent than nuclei of adjacent mesoderm cells (asterisks); scale bar 10 μm. F. Detail of stomodaeum in cross section, stomodaeal lumen (st) surrounded by epithelial cells (ste); note that some epithelial cells contain numerous yolk granules (yg); scale bar 3 μm. G. Section posterior to prototroch, slightly oblique section plane; first tier of posttrochal cells (pt1) constituting outermost layer of apical cap; asterisk marks stomodaeal opening; scale bar 20 μm. H. Detail of protonephridial duct (pdu) opening into cleft between trunk (tr) and apical cap (ac); scale bar 2 μm. I. Terminal cell (tc) with ultrafiltration weir (arrows marking two pores) and protonephridial duct (pdu); note cilia and microvilli in the duct lumen; scale bar 1 μm.

The larval body is composed of a bell-shaped apical cap and a distinct, continuously growing trunk (Fig. 4A, Fig. 5A-D). In larvae seven days after hatching all apical cap cells contain numerous yolk granules (Fig. 6A, D-G). Some granules show a distinct layering with a dense core and outer concentric layers of differing electron density (Fig. 6C). Cerebral depressions can be identified anterior to the prototroch (Fig. 6A, B): Ectodermal nervous tissue with a microvillous surface lines a pair of short, tube-shaped invaginations (Fig. 6C). There is a single pair of longitudinal neurite bundles in the trunk and some neurites originating in the apex of the apical cap, which probably are associated with the apical organ (Fig. 5B). Large unicellular mucus glands are opening between pretrochal cells (Fig. 6A). The larvae have a well-developed ciliated stomodaeum (Figs. 5B, D, 6D, F) that opens into the ring-shaped furrow between apical cap and trunk (Fig. 6G) and ends blindly. An oral ciliary band confluent with the stomodaeal ciliation surrounds the entire anterior trunk (Fig. 5B, C). This band is completely disguised by the test and thus not obvious in surface views. At that stage, the midgut is not yet developed and the central part of the trunk contains a cluster of cells with numerous yolk granules (Fig. 6G). A pair of protonephridia is located on both sides of the stomodaeum (Fig. 5B). Each protonephridium is composed of a single ciliated terminal cell with a simple ultrafiltration weir (Fig. 6I), several ciliated duct cells, and opens via a single nephropore cell into the furrow between test and trunk (Fig. 6H). The cells covering the inner surface of the apical cap are very similar in ultrastructure to cells lining the trunk. Most of the trunk surface is covered by microvilli and a few single cilia that gradually become embedded into a cuticular layer. There are conspicuous depressions, probably epidermal glands, scattered over the dorsal and lateral trunk surface (Fig. 4A, B). A broad ciliary band marks the ventral longitudinal midline, the future foot (Fig. 4A). The ventral cilia are spread apart and there is no distinct boundary between this "foot" and the adjacent trunk surface (Fig. 4B). A ring-shaped telotroch surrounds the posteriormost trunk cells that bear a tuft of long compound cilia.

#### Day 8-9 after hatching

After day seven, the larval test begins to degenerate. The post-trochal test cells lose their regular quadrangular shape and diminish in size and number (Fig. 4C). It appears that they gradually get detached from the apical cap by epidermal trunk tissue that is protruding below them. The posttrochal cell tiers show a ventral cleft (Fig. 4A). At least in some specimens the ventral part of the prototroch appears more strongly developed and may, together with the ventral ciliary band, serve for creeping on the substratum (Fig. 5C, D).

#### Day 10-16 after hatching

**Figure 7:**
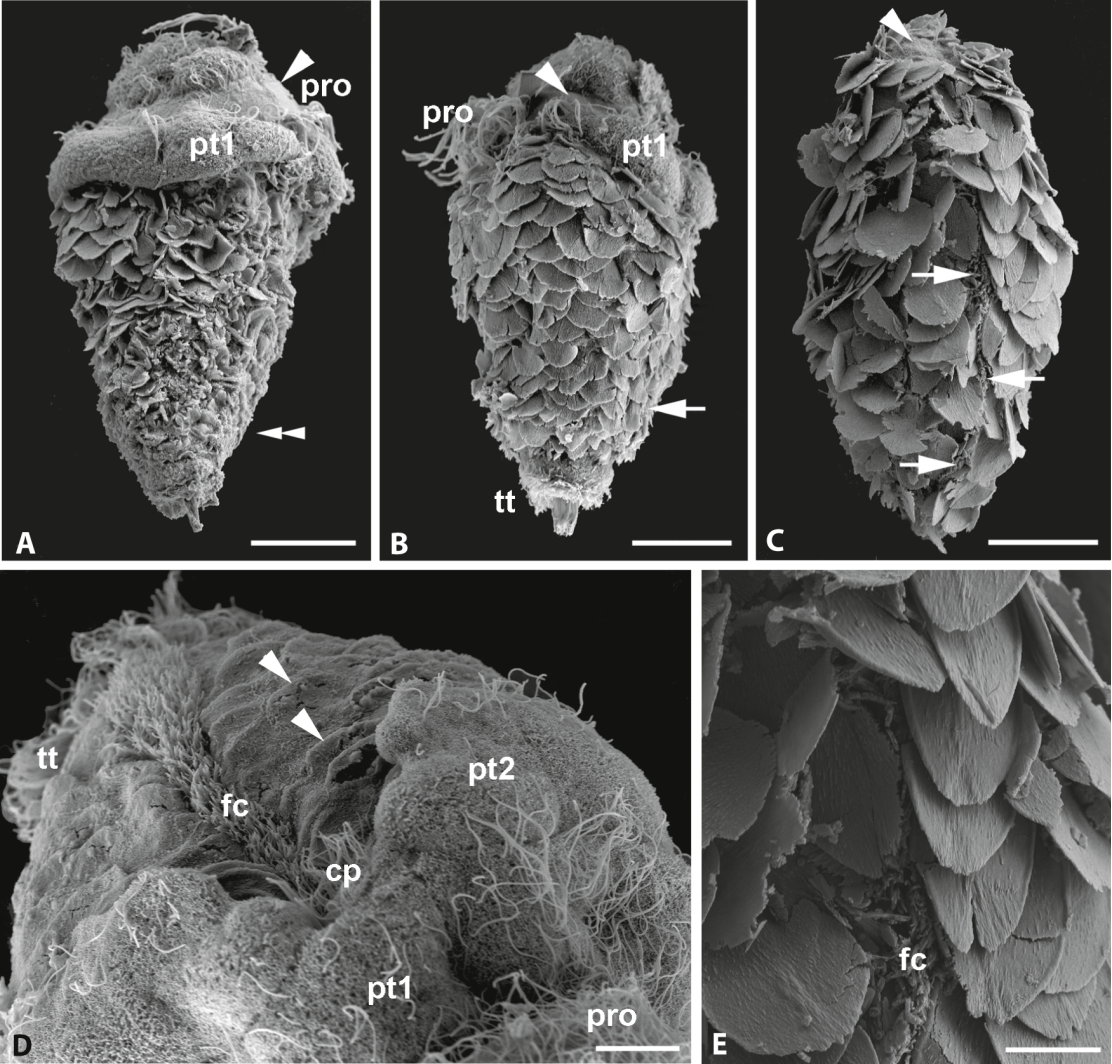
***Wirenia argentea *larvae and postlarvae (days 10-16), SEM micrographs**. A. Day 10 larva in dorsolateral view with apical cap consisting of pretrochal cells, prototrochal cells (pro) characterized by cilia-free surface (arrowhead), and first tier of posttrochal cells (pt1); note that sclerites covering the posterior trunk are still partly covered with cuticle (double arrowhead); scale bar 30 μm. B. Day 12 larva in lateral view with fully developed postlarval sclerites; prototrochal cilia (pro), some cells of first tier of posttrochal cells (pt1) and telotroch (tt) still present; arrow marks foot; scale bar 30 μm. C. Day 16 larva in ventral view, telotroch and most of apical cap reduced; note a last prototrochal cell (arrowhead) and some compound cilia on the anterior body tip; arrows mark foot; scale bar 30 μm. D. Day 10 larva in an apical/ventral view showing ventral cleft between second tier posttrochal cells (pt2) exposing part of ciliary pit (cp); note that epidermal sclerites are still covered by cuticle (arrowheads); fc: foot cilia, pro: prototroch cilia, pt1: first tier posttrochal cells, tt: telotroch; scale bar 10 μm. E. Detail of C, epidermal sclerites flanking and partly obscuring foot cilia (fc); scale bar 10 μm.

The first epidermal sclerites are formed under the cuticle (Fig. 7D). We could observe considerable heterochrony in scleritome development: some specimens were covered in sclerites while still bearing posttrochal cells, while in others the sclerites appeared much later (compare Fig. 5D with, Fig. 7A, B, D). The sclerites are simple, flat scales, measuring between 40 × 15 μm and 55 × 25 μm. They are densely and regularly arranged over the whole body, similar to shingles on a roof, and the posterior sclerites emerge slightly later than the anterior ones (Fig. 7A-C). These larval/postlarval sclerites are distinctly different in shape from the sharply pointed adult sclerites that are characterized by a distinct median keel.

**Figure 8:**
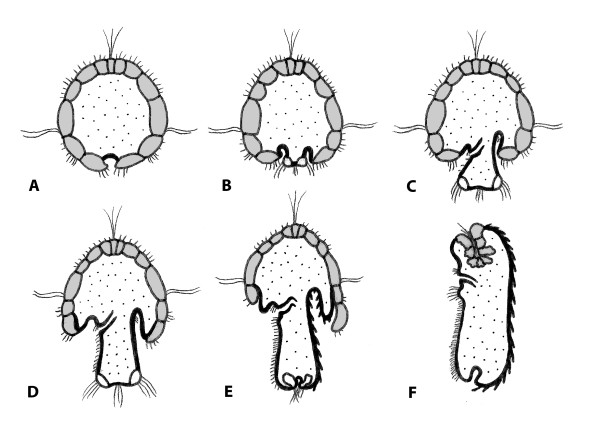
***Wirenia argentea *ontogeny - schematic representation of the development of test and epidermis**. Lateral view, ventral side to the left; test cells grey, other ectdodermal cell layers black, mesodermal tissue dotted (inner organs not depicted). A. Day 1 larva. B. Day 3 larva with trunk rudiment. C. Day 4 larva with short trunk. D. Day 7 larva, note dense ventral ciliation (foot) and ingression of non-test ectodermal tissue (epidermis) below posttrochal cells. E. Day 10 larva with one of the ventral posttrochal cells shed and remaining posttrochal cells sitting on top of epidermal material; note inversion of telotroch and dorsal sclerites. F. Day 14 postlarva, posttrochal cells shed, pretrochal cells covered by epidermis and largely absorbed.

With ongoing degradation of the posttrochal cells, first the ciliary pit, then the ciliated area surrounding the stomodaeal opening (Figs. 4D, E), and finally the oral trochus (Fig. 5E) become exposed. This is paralleled by an eversion of the apical cap rim, resulting in the smaller ectodermal cells lining the inner surface of the cap to form the outer surface of the anteriormost part of the trunk. Prototrochal, pretrochal, and telotroch cells become gradually reduced, most probably absorbed (Fig. 8E, F). Finally, the postlarval sclerites obscure last remainders of test cells and telotroch (Fig. 7C). The prepedal ciliary pit bears a tuft of compound cilia while the now well defined foot is covered by simple cilia that are very densely spaced (Figs. 4E, 5E). The metamorphosing animals have a single pair of longitudinal neurite bundles that is located laterally (Fig. 5E).

#### Day 17-42 after hatching

At days 17-19, larval organs (including protonephridia and stomodaeal ciliation) are reduced, but the midgut is not fully developed yet. The cilia surrounding the oral opening are reduced and the ciliated stomodaeum is replaced by a non-ciliated pharynx. Cilia visible more posteriorly in the gut of a juvenile 30 days after hatching probably are part of the ciliary tract of the midgut (Fig. 5F). Anterior to the mouth opening the vestibulum is formed, endowed with bundles of sensory cilia (Fig. 5F). In a specimen sectioned at 42 days after hatching the yolk reserves have almost completely been absorbed. Inner organs, including radula, midgut, and sensory structures such as the pedal commissure sac, are well developed. The gonopericardial system is still entirely lacking and we could not detect a heart, pericardium, or related structures. It appears that hindgut and midgut have not yet fused at this stage of development.

## Discussion

### Fertilization in *Wirenia argentea*

According to the general presence of organs known to be involved in internal fertilization (seminal receptacles, copulatory stylets) and a sperm type characteristic for internal fertilization, most - if not all - Solenogastres species appear to deposit fertilized eggs [24]. Reciprocal copulation is thought to be the general mechanism for sperm transfer [5,25]. The low numbers of sperm in the seminal receptacles of our investigated adult specimens, however, raise an interesting notion. The gonad of sectioned specimens contained numerous ripening and mature sperm cells, but we did not observe any copulation behavior in the laboratory. Self-fertilization is known from various mollusks, most interestingly from certain hermaphroditic opisthobranch slugs with internal fertilization [26]. Even if at this point there is no proof for self-fertilization in *W. argentea*, it is possible that the animals can switch from fertilization via copulation (indicated by presence of sperm in the seminal receptacles) to use of own sperm under stressful conditions, such as laboratory culture.

### Oviposition in Solenogastres

In *Wirenia argentea*, eggs are deposited shortly after fertilization and prior to the first cleavage. Brood protection has been described for other species, such as *Halomenia gravida*, observed to carry larvae in the mantle cavity [27], and *Epimenia babai*, having at least the capacity of retaining eggs and larvae for several days in the mantle cavity [28]. *Wirenia argentea *(present data), *Nematomenia banyulensis *[17], *Neomenia carinata *[29], and *Rhopalomenia aglaophaeniae *[30] deposit uncleaved embryos. Solenogasters lay their eggs in paired batches - correlating to the paired arrangement of spawning ducts - with several days to weeks between deposition events. Only in *E. babai *the eggs of each batch are enclosed in a thick mucus sheath [23], while generally there is only a thin mucus coating surrounding the egg hulls. The number of eggs deposited per batch varies largely between species and appears to be dependent on body size of the parent. In addition, more eggs are produced early in the breeding period [[28]; personal observation]. Large *E. babai *specimens of 20 cm body length deposit up to 100 fertilized eggs at once and up to four such batches within one night [23]. Thompson's single *N. carinata *specimen was 2 cm long and also produced up to 100 eggs per batch [28], while small species such as *W. argentea *deposit only 4-6 eggs per batch. Egg size, however, is not correlated in a linear way to average species size. The largest eggs with a diameter of 700 μm (including egg hull) have been found in *N. carinata *[29], a species with a maximum body size of 3 cm, while the eggs of the up to 20 cm long *E. babai *measure 350 μm [23], and the eggs of both *N. banyulensis *with a maximum body length of 3 cm [30] and *W. argentea *with a body length of about 0.5 cm are 200 μm in diameter. It has to be noted that in some cases (*E. babai*, probably also *N. carinata*) the space between embryo and egg hull is considerably larger than for example in *W. argentea*.

### Hatching and larval behavior in Solenogastres

As described here for *Wirenia argentea*, solenogaster larvae usually hatch at a swimming stage when the prototroch ciliation is well developed [23,28,29]. In *Epimenia australis*, by contrast, the larvae leave the "fertilization membrane" (egg hull) much later, namely at the postlarval crawling stage [24]. Since these observations are based on fixed material only, they require corroboration by *in vivo *studies. In contrast to the negative geotaxis observed in newly hatched larvae of *Wirenia argentea*, *Neomenia carinata *larvae seem to stay within a 2 cm range from the bottom [29]. The basic information available for *Epimenia babai *[23,28] shows similarities to our findings on *W. argentea*. In the former species, the younger larvae exhibit vivid spiral swimming, typical for spiralian trochophore-like larvae, and do not show any phototactic behavior. In *E. babai*, metamorphosis starts at days seven to eight after hatching at 16°C [23]. Beating of the prototroch cilia can still be observed at this point, but the larvae lose their ability to swim before the trunk is covered by sclerites. This is different to our observation on *W. argentea*. Probably the relatively short prototroch cilia of the metamorphosing *E. babai *larvae render swimming more difficult. *Epimenia babai *juveniles lived up to one month after metamorphosis without food uptake [23], which despite of the difference in culturing temperature is similar to *W. argentea*.

### The pericalymma larva of Solenogastres

The larva of *Wirenia argentea *is a pericalymma larva *sensu *Salvini-Plawen [4] or test cell larva *sensu *Heath [27] and Thompson [29], with a pronounced apical cap (preoral test). Okusu proposed homology of the somewhat reduced apical cap of *Epimenia babai *with the larger cap of other species [23] and we agree with this conclusion. In general, the posterior rim of the apical cap of solenogasters forms a ring-shaped fold (see Fig. 1J) and the relatively large cells lining the outer surface are commonly referred to as test cells. The general arrangement of the partly ciliated test cells stays the same until degeneration of the cap sets in. In *W. argentea*, the inner surface of the posterior rim of the apical cap - facing the trunk - is formed by smaller cells similar to epidermal cells of the trunk (Fig. 8A-F). No such information is available for any other solenogaster species. In some, such as *Epimenia babai*, the free part of the posterior apical cap rim is short and is reduced (stretched) at an early larval stage, thus exposing the blastopore [22,23,28]. In others, such as *Wirenia argentea *(described herein), *Nematomenia banyulensis *[17], and *Neomenia carinata *[29], the apical cap is more pronounced and the rim persists until the larva settles. *Rhopalomenia aglaophaeniae *[30] and *Halomenia gravida *[27] appear to present an intermediate stage, even if the status of the latter species is somewhat unsure because the brooded larvae detected in the mantle cavity of a sectioned adult do not represent the entire developmental sequence until metamorphic competence [27]. The fact that *H. gravida *and *R. aglaophaeniae*, that have larvae bearing a well-developed apical cap, and *E. babai *with a reduced cap have been assigned to the supposedly derived order Cavibelonia [1,31] points towards a tendency to cap size reduction within the clade.

### Spiralian test cell larvae

The term "test cell larva" stems from early work on protobranch bivalve development and refers to the cover composed of large cells that entirely enclose the developing larval body [9,10]. Test cells in general are cleavage arrested and deciduous, i.e., they are shed or absorbed during metamorphosis. Salvini-Plawen used the term pericalymma larva to describe a larval type characterized by a test-like cover (calymma) surrounding at least parts of the posterior larval body [7]. He proposed homology of the larval tests between protobranch bivalve and solenogaster larvae, the so-called endolarva of polygordiid and oweniid polychaetes, and the sipunculan "serosa" larva. He furthermore suggested this lecitotrophic larval type to represent a more ancestral condition than the planktotrophic trochophore or veliger larvae [see also [11]]. There are, however, some conspicuous morphological differences between the diverse pericalymma larvae. The protobranch test is a preoral layer of large ectodermal cells enveloping the entire larval body. In older larvae it is separated from underlying tissues by a non-cellular space and thus only loosely connected to the remaining body [10,32,33]. Upon metamorphosis, this entire layer of test-cells is shed. Similar to the situation in Solenogastres, the apical-most cells are absorbed and in Nuculoida there is a ciliated postanal organ somewhat comparable to the posteriormost trunk area in Solenogastres [33]. As an important difference to Solenogastres, the ecdodermal cell layer developing under the protobranch test forms the adult mantle epithelium and shell without any evertion process during metamorphosis. The protobranch test is entirely ciliated or bears three ciliary bands, but never has a single distinct locomotory band ("prototroch").

The overall morphology of larval test and ciliation is astonishingly similar in polychaete endolarvae and in solenogaster pericalymma larvae. In the first, however, the test cells are located preorally and postorally and also anterior and posterior to the prototrochal ciliary band, while in the latter all test cells are preoral and the distance between prototrochal cilia and oral opening is large. In general a metatroch appears to be lacking in mollusks (see below). The serosa or cellular embryonic envelope of the *Sipunculus nudus *(Sipuncula) larva appears to be a peculiarity of this single species [34,35], and already Gerould proposed it to be secondarily derived from prototrochal cells [36]. Based on morphological differences and considering the fact that according to recent phylogenies the serosa- and endolarvae do not occur in supposedly "basal" branches of Sipuncula and Polychaeta, it appears more probable that "test cell larvae" have evolved multiple times within Spiralia [see also [12,23]]. Further knowledge about cell progeny is crucial to assess this hypothesis.

The finding of protonephridia in larval *W. argentea *is a novelty for solenogasters. These excretory organs are an important trait because their absence in marine mollusks has been used as one of the main arguments against the homology of annelid and mollusk trochophore larvae [7]. Since then, larval protonephridia have been found in several mollusk taxa including caudofoveates [19] and chitons [37], and the evidence for larval protonephridia in *W. argentea *presented here confirms that these organs are ancestral for Mollusca and Trochozoa/Spiralia in general.

### Trochs and other ciliation in Solenogastres

The apical cap of all hitherto described solenogaster larvae has a well-developed equatorial band of compound cilia, the prototroch *sensu stricto *[17,22,23,28,29]. Heath did not observe any ciliary bands in the brooded larvae of *Halomenia gravida *[27], but this might be due to the state of preservation of his material. In free-swimming solenogaster larvae, compound prototrochal cilia are generally restricted to a single row of cells. The cells of the remaining apical cap bear single cilia, which may be grouped into more or less diffuse bands, as for example in the post-trochal cell tiers of *W. argentea*.

Solenogaster larvae have a densely ciliated ring-shaped zone at the posterior end of their trunk, a telotroch, which is already present in very early larval stages and that prevails until metamorphosis [[23,29], and herein]. It is not known if the compound cilia of the telotroch take part in locomotion. Interesting is the ciliation of the oral area and stomodaeum in *W. argentea *larvae, and the distinct troch surrounding the trunk apically (oral troch). Such ciliation has not been described before. Adult solenogasters have a preoral ciliary band surrounding the sensory vestibulum but in general lack locomotory cilia at the mouth opening and in the pharynx [38,39]. The dense oral and stomodaeal ciliation in *W. argentea *larvae (especially in the earlier stages) could thus tentatively be interpreted as rudiments of a former planktotrophic feeding apparatus. The oral troch of *W. argentea *is located in a position comparable to the adoral ciliary zone or even to a metatroch in planktotrophic trochophore larvae (see below). In the recent, definitely non-feeding *W. argentea *larvae, the oral troch might serve to ventilate the ring-shaped cavity between the apical cap and the anterior trunk. The pericalymma larvae of Solenogastres in general are thought to be non-feeding, i.e. lecitotrophic [4], but in *N. carinata *Thompson shows the gut to be complete already at a very early stage [see figure 22 in reference [29]]. If these observations are correct, swimming stages or at least very young postlarvae of *N. carinata *may be capable of feeding. His text, however, diverges from the figures, because he describes the fusion between midgut and hindgut to take place during metamorphosis [29]. This is at an early stage, compared to e.g., *W. argentea *or *Epimenia babai *[22,28], but fits the general developmental patterns in lecitotrophic mollusk larvae [40].

The foot of *W. argentea*, similar to *Nematomenia banyulensis *[29] and *E. babai *[23], arises as a simple ciliary band ventrally on the trunk of the swimming larva and only later develops into a longitudinal ciliated fold that may be protruded or retracted by means of dorsoventral body muscles. Generally, the solenogaster foot - in contrast to the muscular foot of chitons and conchiferan mollusks - lacks intrinsic musculature and locomotion is largely due to ciliary action [[25] and own observations]. As stated for *E. babai *[28], there is no indication in development that the solenogaster foot represents a reduced muscular foot.

### Homology of trochs in spiralian larvae

Cell lineage studies could confirm the homology of the prototroch across diverse spiralians, including chitons, polychaetes, and nemerteans [41-43]. Typically, prototrochal cells become cleavage arrested early in development, are thus larger than regularly dividing cells, and differentiate as ciliated cells [43]. The definitions of prototroch cells and larval test cells thus are quite similar (see above). There is little doubt that the main locomotory troch in solenogaster larvae represents a prototrochal ciliary band comparable to that, for example, of gastropod veliger larvae or polychaete trochophores, but this prototroch *s. str*. of mollusks is merely locomotory in function [12]. Information about cell progeny is necessary to infer if and to which degree the larval test of solenogasters and protobranch bivalves represents an enlarged prototrochal area. Cell lineage studies could also shed light on the potential homology of the oral troch of *W. argentea *to the adoral ciliary zone of other lophotrochozoan larvae or to a metatroch. It shall be noted here that the so-called "metatroch" of the gastropod *Crepidula fornicata *is probably not homologous to the metatroch of annelid trochophores [44,45]. The ventral ciliation of the *W. argentea *larva - the future foot - resembles the early foot ciliation in gastropod and bivalve larvae, but also the locomotory neurotroch (gastrotroch) of certain polychaete trochophores [12,45]. It is tempting to propose an evolutionary pathway leading from such larval ventral ciliation via a pedal fold, such as present in recent solenogasters, to the more complex muscular foot of "higher" mollusks.

### Development of epidermal hard parts in mollusks

Our results on epidermal sclerite development demonstrate that in *W. argentea *dorsal sclerites do not show a serial arrangement in any ontogenetic stage. Pruvot's description of seven dorsal rows of imbricating scales in *N. banyulensis *[17] thus remains an isolated observation based on a single specimen. The situation described for an unidentified solenogaster postlarva [18] is different in that no single rows of sclerites were present, but an arrangement the authors termed "iterated dorsal groups of spicules". These six groups of sclerites appeared separated by bare areas that were proposed to be homologous to polyplacophoran shell fields. The entire sclerite arrangement of the postlarva was compared to the scleritome of Cambrian fossils, such as *Wiwaxia corrugata *and *Halkieria evangelista*. Together with this remarkable specimen, a second and slightly larger representative, supposedly of the same species, lacking the bare areas was found. The presence of very characteristic solid hook-shaped sclerites [see figure 1 in reference [18], photograph of the small postlarva] suggests an affinity to the probably derived solenogaster clade Phyllomeniidae [31]. This phylogenetic status of the postlarvae somewhat weakens the evolutionary arguments presented [18], but the findings are of interest when considering the ontogeny of the scleritome (sclerite armour) in solenogasters. The merely 0.4 mm long postlarva had a stunning diversity of sclerite types and the sclerites were quite large in size compared to the total body length, as common in juvenile solenogasters [46].

It has been known for a long time [23,30] that in representatives of the supposedly derived clade Cavibelonia the hollow acicular adult sclerites are preceeded by scale-like larval sclerites. The presence of scale-like sclerites has been interpreted as the ancestral status, thus rendering the order Pholidoskepia, characterized by a merely scaly scleritome, the most "basal" clade [5,31]. Here, we show that also in Pholidoskepia species, such as *W. argentea*, the adult scales have larval precursors that are different in shape. This is an interesting analogous (?) case to shell development in gastropods with embryonic *versus *adult shells. In chiton larvae no such ontogenetic change in sclerite types is known and the mantle sclerites arise slightly earlier than the first seven shell plates, even though the shell plate anlagen are obvious as dorsal grooves already earlier than the sclerites [24,47-50]. The sclerite and shell plate producing cells are of a more diverse and partly different cell progeny as compared to the shell gland cells of conchiferan mollusks: Conchiferan shells stem predominantly from the 2d and to a lesser extent from the 2a, 2b, 2c and 3c micromeres, while the spicule-producing cells of the chiton *Mopalia mucosa *stem from the 1a, 1d, 2a, 2d, and 3a micromeres and shell plates from the 2d, 3c, and 3d micromeres [41]. In chiton larvae the most anterior part of the sclerite-bearing girdle appears anterior to the prototroch [41,49] while in solenogaster larvae the cuticle- and sclerite-bearing epidermis is restricted to the area posterior to the main locomotory cilary band. Data on the progeny of solenogaster sclerite producing cells are necessary for further interpretation of this difference.

## Conclusions

Solenogastres (Neomeniomorpha) develop via a trochophore-like lecitotrophic larva with a pronounced preoral apical cap covered by large, cleavage-arrested deciduous cells. These cells have previously been termed test cells and appear at least partly to represent an enlarged prototrochal area. The morphology of the solenogaster apical cap differs considerably from the morphology of the so-called larval test of other spiralian larvae, namely of protobranch bivalves and selected polychaetes and sipunculans, and thus a homology of test-cell larvae (pericalymma larvae) across spiralian clades appears unlikely. The presence of an oral troch encircling the larval body and covered by the apical cap as well as the dense ciliation of the non-functional stomodaeum in the *W. argentea *larva may point towards an earlier function in feeding and might indicate secondary lecithotrophy in solenogaster larvae. Characters linking the solenogaster larva to other spiralian trochophores are the presence of paired larval protonephridia and the ventral trunk ciliation that resembles a locomotory neurotroch in certain polychaete trochophores and later develops into the ventral creeping sole (non-muscular foot) typical for solenogasters. Differences to other spiralian trochophores include the long distance between the prototroch and the stomodaeal opening and the very late appearance of the mid- and hindgut in postlarval stages. The dorsal trunk cuticle with calcareous sclerites is an adult character that generally arises early in solenogaster development. In *W. argentea*, the sclerites are evenly distributed and no serially arranged calcareous structures comparable to those found in polyplacophoran larvae are present. Our results provide no evidence for promoting a chiton-like ancestor for recent mollusks, but rather support an ancestral position of Solenogastres.

## Methods

### Animal collection and cultures

About 300 specimens of adult *Wirenia argentea *Odhner, 1921, syn. *Aestoherpia glandulosa *Salvini Plawen, 1985, were collected using an environmental sledge (RP sledge) in Hauglandsosen (Bergen, Norway), at 180-220 m deep. The animals were extracted from the sediment samples immediately after collection and were kept in 50 ml plastic jars with 20-30 specimens per jar at 7°C in constant darkness; light exposure occurred only during handling. During the first week, 2/3 of the seawater was changed and embryos and larvae collected every day, after the first week every second day. Adults did not feed, even though we offered a number of potential prey organisms (diverse cnidarians). The animals started to deposit eggs several hours to days after capture and continued to do so for nearly 2 months. For timing of development, embryos were transferred to similar plastic dishes with filtered seawater directly after deposition. Timing is based on ca. 40 individuals. Additional larvae (ca. 200) were transferred to clean dishes at the earliest swimming stage. Postlarvae were kept to a maximum of 42 days after hatching. Larvae and postlarvae were relaxed by drop-wise addition of cold 7.14% MgCl_2 _and fixed appropriately (see below).

### Scanning electron microscopy

Specimens were fixed 1.5 h on ice in 1% OsO_4 _in filtered sea water, dehydrated in a graded ethanol series, critical point dried, and sputter coated with gold-palladium. Digital images were acquired using a ZEISS Supra 55VP scanning electron microscope. About 40 specimens were studied.

### Histology and transmission electron microscopy

Specimens were fixed for 12 hours at 7°C in 4% glutaraldehyde in 0.2 M sodium cacodylate buffer (0.1 M NaCl, 0.35 M sucrose at pH 7.3) and postfixed for 1.5 h on ice in 1% OsO_4 _in filtered sea water. The adult specimens were decalcified for 3 h at room temperature in a 3% solution of ascorbic acid in distilled water. All specimens were dehydrated in a graded ethanol series followed by 100% propylene oxide and embedded in Agar Low Viscosity Resin (Agar Scientific, UK). Semithin serial sections (1 or 2 μm) were produced on a Reichert Ultracut S microtome using a Diatome Histo Jumbo Knife [51] and stained with toluidine blue. Digital images were produced using a CCD microscope camera (Spot Insight, Diagnostic Instruments Inc., USA) on a Leica DMB-RBE microscope. Serial sections of six adult specimens and four larvae were produced. Ultrathin sections were cut using the same microtome, mounted on copper slot-grids, contrasted with uranyl acetate and lead citrate, and examined with a Morgagni 268 electron microscope. Digital images were produced with a Mega View III Soft Imaging System and edited using Adobe Photoshop CS2 9.0.2 and Adobe Illustrator CS11. Our results on internal ultrastructure are based on two larvae and two postlarvae of different stages.

### Immunocytochemical labeling, nuclear staining, and confocal microscopy

Larvae were fixed in 4% paraformaldehyde (PFA) in 0.1 M phosphate buffered saline (PBS; pH 7.3) for 4-12 h on ice. After fixation, the samples were washed twice for 15 min in PBS and stored at 4°C in PBS with 0.1% NaN_3 _added. Specimens were permeabilized for 1 h in PBS containing 1% Triton X-100 (PTA) at room temperature. Blocking of unspecific binding sites was carried out using a solution of 6% normal goat serum (Jackson Immunoresearch, West Grove, Pennsylvania, USA) in PTA (block-PTA) for 3 h at room temperature. Antibodies raised in mouse against acetylated α-tubulin (Sigma, Steinheim, Germany) were used in a 1:500 dilution in block-PTA. The specimens were incubated for 20-24 h at room temperature, followed by four washes in block-PTA over 3 h at room temperature or overnight at 4°C. Subsequently, they were incubated in a 1:200 dilution of a FITC-conjugated goat anti-mouse secondary antibody (Sigma) in block-PTA for 20-24 h at room temperature, followed by four washes over 3-4 h in PBS at room temperature. Some specimens were consecutively labeled for 30 min with the Hoechst 33342 nuclear stain (Molecular Probes, Eugene, Oregon, USA). Specimens were mounted in Fluoromount G (Southern Biotech, Birmingham, Alabama, USA). Image acquisition was performed on a Leica DM IRBE microscope equipped with a Leica TCS SP 2 confocal laserscanning unit. Z-projections of whole-mount preparations were obtained. The confocal images herein represent projection images produced by merging stacks of optical sections.

## Competing interests

The authors declare that they have no competing interests.

## Authors' contributions

CT designed the study, carried out laboratory work, and wrote the manuscript. AW contributed to CLSM work, interpretation of data, discussion of results, and improved the text. All authors read and approved the final manuscript.
